# Large-scale asymmetric introgression of cytoplasmic DNA reveals Holocene range displacement in a North American boreal pine complex

**DOI:** 10.1002/ece3.294

**Published:** 2012-07-06

**Authors:** Julie Godbout, Francis C Yeh, Jean Bousquet

**Affiliations:** 1 Canada Research Chair in Forest and Environmental Genomics, Centre for Forest Research and Institute for Systems and Integrative Biology, Université Laval1030 avenue de la Médecine, Québec, Québec, Canada, G1V 0A6; 2 Department of Renewable Resources, Faculty of Agricultural, Life and Environmental Sciences, University of AlbertaEdmonton, Alberta, Canada, T6G 2P5

**Keywords:** Chloroplast DNA, hybrid zone, mitochondrial DNA, phylogeography, *Pinus banksiana*, *Pinus contorta*

## Abstract

Jack pine (*Pinus banksiana*) and lodgepole pine (*Pinus contorta* var. *latifolia*) are two North American boreal hard pines that hybridize in their zone of contact in western Canada. The main objective of this study was to characterize their patterns of introgression resulting from past and recent gene flow, using cytoplasmic markers having maternal or paternal inheritance. Mitochondrial DNA (mtDNA) and chloroplast DNA (cpDNA) diversity was assessed in allopatric populations of each species and in stands from the current zone of contact containing morphological hybrids. Cluster analyses were used to identify genetic discontinuities among groups of populations. A canonical analysis was also conducted to detect putative associations among cytoplasmic DNA variation, tree morphology, and site ecological features. MtDNA introgression was extensive and asymmetric: it was detected in *P. banksiana* populations from the hybrid zone and from allopatric areas, but not in *P. contorta* populations. Very weak cpDNA introgression was observed, and only in *P. banksiana* populations. The mtDNA introgression pattern indicated that central Canada was first colonized by migrants from a *P. contorta* glacial population located west of the Rocky Mountains, before being replaced by *P. banksiana* migrating westward during the Holocene. In contrast, extensive pollen gene flow would have erased the cpDNA traces of this ancient presence of *P. contorta*. Additional evidence for this process was provided by the results of canonical analysis, which indicated that the current cpDNA background of trees reflected recent pollen gene flow from the surrounding dominant species rather than historical events that took place during the postglacial colonization.

## Introduction

The impact of Quaternary glacial cycles on the distribution of species and their genetic diversity is now well documented. Indeed, the modern population genetic structure of several species currently found at temperate and boreal latitudes still carry the signature of vicariance induced by their retreat in isolated refugia (for reviews, see Hewitt 2004; Soltis et al. 2006; Jaramillo-Correa et al. 2009; Shafer et al. 2010). At the interspecific level, these migrations likely resulted in repeated hybridization events between interfertile species in spatially and temporally dynamic zones of contact. Thus, genetic footprints of historical contact are expected to be found in presently allopatric areas, far from current cross-species gene influence (Bouillé et al. 2011).

Anderson and Hubricht (1938) defined introgression as the result of the incorporation of genes from one species into another via hybridization and through repeated backcrossing of hybrids with the parental species. From an evolutionary perspective, the role of introgression is not fully understood. One benefit previously suggested is an increase in intraspecific genetic diversity, and thus, of raw material on which natural selection could possibly act on (Anderson 1949). However, although introgression can be easily detected using molecular or morphological markers (Klier et al. 1991), the significance of its role in adaptation is much more difficult to demonstrate (Rieseberg and Wendel 1993; Arnold 2004; but see also Anderson et al. 2009; Whitney et al. 2010). Modeling has also shown that more neutral factors such as the direction and amplitude of gene flow can be key factors in shaping introgression patterns (Currat et al. 2008; Meirmans et al. 2009). From a biogeographic perspective, investigating modern geographic patterns of introgression could also provide insight into historical gene exchanges in species complexes and how natural ranges might have evolved and fluctuated in the recent past.

In Pinaceae, mitochondrial and chloroplast genomes are inherited maternally (Dong and Wagner 1993) and paternally (Neale and Sederoff 1988), respectively. Hence, using markers from both genomes allows tracking gene exchange transiting via seeds only (mitochondrial DNA [mtDNA]) or pollen and seeds (chloroplast DNA [cpDNA]). These contrasted modes of dispersion usually lead to differential gene flow (Petit et al. 2005), resulting in different mtDNA and cpDNA patterns potentially revealing cytoplasmic genome capture events among species (e.g., in trees: Tsumura and Suyama 1998; Senjo et al. 1999; Du et al. 2009, 2011; Bouillé et al. 2011) or among genetically distinct intraspecific lineages (Gérardi et al. 2010; Wei et al. 2011). Moreover, such asymmetric gene dispersion is thought to affect the direction and rate of cytoplasmic introgression (Currat et al. 2008; Du et al. 2009, 2011).

**Figure 1:**
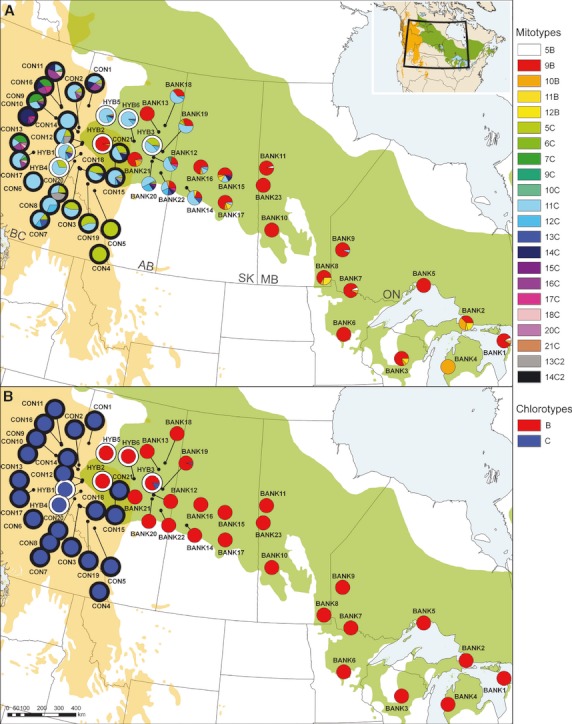
Geographic distribution and frequency of mitotypes (a) and chlorotypes (b) observed in 50 *Pinus contorta* and *Pinus banksiana* populations. Pie charts highlighted by black circles correspond to *P. contorta* populations, those by white circles to hybrid populations and the other ones to *P. banksiana* populations. Orange background, the natural range of *P. contorta*; green background, that of *P. banksiana* (according to Little 1971). Abbreviations of Canadian provinces: AB, Alberta; BC, British Columbia; MB, Manitoba; ON, Ontario; SK, Saskatchewan. (a) Mitotypes are coded with a first number corresponding to the number of repeats observed at locus *nad7* intron 1, followed by a letter and a possible other number indicative of a predominantly *banksiana* type (B) or *contorta* type (C or C2) according to the polymorphism observed at locus *nad1* intron b/c. (b) Chlorotype B was defined as *banksiana* type and chlorotype C, as *contorta* type.

Jack pine (*Pinus banksiana* Lamb.) and lodgepole pine (*Pinus contorta* Dougl. ex. Loud.) belong to subgenus *Pinus* and sub-section *Contortea* along with *Pinus virginiana* and *Pinus clausa*. They are the only two boreal pines in North America (Critchfield and Little 1966). The combined natural ranges of *P. contorta* and *P. banksiana* extend from the Pacific to the Atlantic coasts and cover the entire North American boreal region (Fig. 1). *Pinus contorta* has a typically latitudinal distribution in the Pacific Northwest, whereas *P. banksiana* rather stretches longitudinally from western Canada to the Atlantic Maritimes region in eastern Canada (Critchfield and Little 1966). *Pinus contorta* comprises four subspecies (ssp. *latifolia, contorta, murrayana,* and *bolanderi)*. Subspecies *contorta* and *bolanderi* are found along the Pacific coast and *murrayana* in the Sierra Nevada. Subspecies *latifolia* extends eastward from the interior of British Columbia (BC) to Alberta where its distribution overlaps with that of *P. banksiana*.

Hybrid individuals presenting intermediate morphological features between *P. contorta* ssp. *latifolia* and *P. banksiana* have been frequently reported in the zone of contact found in western and northern Canada (Rudolph and Laidly 1990; Fig. 1). The zone located in western Alberta has been extensively studied using morphological (Moss 1949; Critchfield 1957), biochemical (Zavarin et al. 1969; Pollack and Dancik 1985), and genetic markers (Wagner et al. 1987; Wheeler and Guries 1987; Wagner et al. 1991; Dong and Wagner 1993; Ye et al. 2002). However, little is known about the hybridization dynamics and the role of Holocene postglacial migrations in shaping introgression patterns between these species. Indeed, although these studies contributed to gain an understanding of gene exchange in this species complex, their geographically limited coverage provided an incomplete picture of introgression patterns at both temporal and spatially large scales.

The geographic distribution of mtDNA and cpDNA diversity was previously analyzed in *P. banksiana* and *P. contorta* (including all of its four subspecies) to explore their postglacial histories. No clear geographic structure was detected using cpDNA markers in either species (Marshall et al. 2002; Godbout et al. 2010). In contrast, mtDNA data showed that both pines had colonized their current natural ranges from multiple and genetically differentiated glacial populations (Godbout et al. 2005, 2008). At least two distinct mtDNA lineages were detected in *P. contorta* ssp. *latifolia* (out of four to five lineages identified in *P. contorta* [Godbout et al. 2008]), whereas at least three mtDNA lineages were identified in *P. banksiana* (Godbout et al. 2005, 2010). A typical mitotypes were also found in one population of *P. banksiana* in eastern Alberta (Godbout et al. 2005), suggesting possible introgression at the far east of the known zone of interspecific hybridization with *P. contorta* ssp. *latifolia*.

The first objective of this study was to assess the extent of cytoplasmic introgression between *P. contorta* ssp. *latifolia* and *P. banksiana* in their current zone of contact in western Alberta and in adjacent parts of their natural ranges (central BC through central Canada) using markers from the differentially dispersed mtDNA and cpDNA genomes. In doing so, we wanted to test if long-range introgression indicative of historical species displacement was present and if it was asymmetric. These results were examined in the light of previously inferred species postglacial histories (Godbout et al. 2005, 2008), and in relation to predictions regarding the direction and intensity of introgression using markers experiencing different rates of gene flow (Currat et al. 2008; Du et al. 2011). The decoupling observed between mtDNA and cpDNA introgression patterns was further assessed from an ecological perspective and in relation to tree morphological classification, with the aim to determine whether introgression was driven by determinants at the local microgeographic scale including the local abundance of the different species.

## Materials and Methods

### Sampling and polymerase chain reaction (PCR) amplification

A total of 925 trees were sampled from 50 populations identified as *P. banksiana*, *P. contorta* var. *latifolia* (hereafter designated more simply as *P. contorta*)*,* or hybrid (see below for criteria used to designate hybrid populations). Nineteen of these populations (*P. banksiana* = 14 [*n* = 226]; *P. contorta* = 5 [*n* = 50]) originated from two provenance tests managed by the Ministère des Ressources naturelles et de la Faune of Québec, where each provenance was established from seeds harvested from 8 to 100 trees per stand, as previously described (Godbout et al. 2005, 2008). Additionally, 17 populations were obtained from the National Tree Seed Centre (Fredericton, New Brunswick, Canada; *P. banksiana* = 5 [*n* = 77]; *P. contorta* = 12 [*n* = 182]). These populations were sampled in provenance trials where each provenance was established from seed harvested from a minimum of 20 trees per stand. Furthermore, 14 populations (hereafter named “in situ sampling”) were sampled and analyzed in the zone of sympatry and adjacent areas in western Alberta. These in situ populations were selected to be representative of the various morphological population types encountered in the zone of contact, with six populations (*n* = 169) labeled as hybrid (each with at least 90% of their trees classified as morphological hybrids), four populations (*n* = 109) labeled as *P. banksiana*, and four populations (*n* = 122) labeled as *P. contorta*. Both *P. banksiana* and *P. contorta* in situ populations presented a maximum of 5% of morphological hybrids. Species or hybrid identification was based on morphological characters (growth form, cone shape and orientation, foliage, and branching habits) according to criteria proposed by Rudolph and Yeatman (1982). Ecological classification (ecoregion type and ecosite type, as described in Beckingham et al. 1996) was also determined for each of these 14 in situ populations. DNA was extracted from frozen needles, embryos, or young seedlings. DNA extraction was performed with the Dneasy Plant Mini Kit (Qiagen; Mississauga, Ontario, Canada) following the manufacturer's instructions.

The screening of mtDNA and cpDNA polymorphisms was conducted on an exploratory panel combining 12 individuals of each species. These trees were selected from populations located far from the reported hybrid zone, so as to avoid hybrid or introgressed individuals. The main goal was to identify polymorphisms that were species-specific for each cytoplasmic genome. In addition, a hyper-variable mtDNA marker was sought to identify the geographic origin of mtDNA introgression (see Results). In total, 29 mtDNA regions (Demesure et al. 1995; Jeandroz et al. 2002; Jaramillo-Correa et al. 2003; Godbout et al. 2005) and 19 cpDNA regions (Taberlet et al. 1991; Wang et al. 1999) were amplified following published PCR protocols in these studies. All loci yielding a single amplification product were then sequenced for both strands with a Sequenase GC-rich kit (Applied Biosystems, Cleveland, Ohio) using the dideoxynucleotide chain termination procedure on an ABI-3130xl (Applied Biosystems) to detect polymorphism among the two pine species. For mtDNA genes, the intron b/c of *nad1* (Demesure et al. 1995) showed an interspecific polymorphism and the intron 1 of *nad*7 (Godbout et al. 2005) showed a hyper-variable intraspecific polymorphism. Internal primers were developed for the *nad1* intron b/c region (internal-*nad*1(b/c)_F: 5′GATCGATCCATAGTGCCGTAA3′ and internal-nad1(b/c)_R: 5′GGGTGCCGCAAGGTTATAC3′; annealing temperature = 60°C). The *nad7* intron 1, whose fragment length polymorphism is caused by a variation in the number of a minisatellite-like repeat, had already been used in previous studies of *P. banksiana* and *P. contorta* populations (Godbout et al. 2005, 2008, 2010). For cpDNA, the *mat*K1, *rbc*L2, *rbc*L3 (Wang et al. 1999), and *trn*L-*trn*F (Taberlet et al. 1991) regions harbored polymorphisms that were species-specific. The internal *nad1* (intron b/c), *mat*K1, *rbc*L2, *rbc*L3, and *trn*L-*trn*F PCR products were, respectively, used in combination with *RsaI, HhaI, SspI, HinfI,* and *MfeI* restriction endonucleases (New England BioLabs, Pickering, Ontario, Canada) following the manufacturer's instructions, and electrophoresed on 2% agarose gels to visualize DNA polymorphisms. Thus, except for the fragment length polymorphism of *nad7* intron 1 directly detectable on 2% agarose gel, all other polymorphisms were revealed using a PCR-RFLP (restricted fragment length polymorphism) assay. Each variant observed on agarose gel was further sequenced to confirm the nature of polymorphism and detect possible cases of fragment length homoplasy.

### Diversity indices and analyses

The two detected polymorphic mtDNA loci on one hand, and the four polymorphic cpDNA loci on the other hand, were considered simultaneously to define multi-locus mitotypes and chlorotypes, which resulted in 22 mitotypes and two chlorotypes (see Results). The number of mitotypes or chlorotypes (*nh)*, the total unbiased diversity index (*h*_t_), the mean unbiased diversity index (*h*_m_; both equivalent to the expected heterozygosity, *H*_E_, for diploid data; [Weir 1996]), and the among-population differentiation *f*_st_ index were estimated for the whole dataset, for each morphological group (populations of *P. banksiana*, *P. contorta,* and hybrid populations), and for each mtDNA Bayesian group defined below.

For the more complex mtDNA dataset, a mitotype network was drawn using the software TCS (Clement et al. 2000), setting the maximum number of steps between two connected mitotypes to seven. This network aimed at revealing related mtDNA variants possibly associated to each pine species. A neighbor-joining (NJ; Saitou and Nei 1987) population dendrogram was built using mtDNA data to infer the population structure of each species, as well as to identify those populations presenting intermediate genetic content, which could be interpreted as introgressed. A matrix of population pairwise *f*_st_ was first calculated using the pairwise difference distance method implemented in the software Arlequin v.3.1 (Excoffier et al. 2005) and loaded in the software MEGA v.4.0 (Tamura et al. 2007) to construct the dendrogram. The tree was anchored using mid-point rooting. A spatial Bayesian analysis of population structure (BAPS) aiming to delineate genetically differentiated and spatially coherent groups was carried out on mtDNA data using the “spatial clustering of groups” option implemented in BAPS v.5.4 (Corander et al. 2008). *K*-values ranging from 2 to 10 were separately tested and the value providing the highest *f*_ct_ value (corresponding to the genetic differentiation among clusters) without yielding a singleton group was considered optimal. This method was preferred over the original log-likelihood method because BAPS delineated over 10 distinct mtDNA groups of little historical significance according to previous studies (see Godbout et al. 2005, 2008). A similar population structure analysis was conducted on cpDNA data to compare the patterns of interspecific structuring between cpDNA and mtDNA. The original log-likelihood method was used on cpDNA data to determine the optimal *k*-value.

A canonical redundancy analysis (RDA) was conducted on the 14 natural populations sampled in situ in the zone of contact to test for possible associations among mtDNA or cpDNA genotypes and the morphological classification of trees (*P. contorta*, *P. banksiana,* or hybrid) or site ecological classification following ecoregions (boreal mixed woods, upper foothills, lower foothills) or ecosites (corresponding to species association identified as a1, b1, c1, d1, e1, and f1; see Beckingham et al. 1996 for a description). These three categorical factors were considered as three independent explanatory matrices. The tested null hypothesis assumed the independence of genotypic information and each of the three factors. Main effects of explanatory matrices were tested via Monte Carlo permutation tests. The canonical analyses and statistical tests were performed using the Vegan Community Ecology Package ver. 1.17-2 for R software v. 2.11.0 (Oksanen et al. 2010).

## Results

### Species distribution of genetic diversity

The *nad*7 intron 1 locus exhibited a minisatellite-motif of 32 nucleotides that yielded a total of 15 haplotypes (corresponding to the numeral code in the mitotype name, see the legend in Fig. 1a). Six indels and one single-nucleotide polymorphism (SNP) were found in the *nad*1 intron b/c region, which resulted in three haplotypes among the total population sampling (corresponding to the letter code B, C, or C2 in the mitotype name, see Fig. 1a). Two of these were associated to *P. contorta* (C and C2), whereas the last one was specific to *P. banksiana* (B). The combination of both mtDNA loci generated a total of 22 distinct mitotypes (Table 1 and Fig. 1a). From the 14 mitotypes detected among *P. contorta* populations, five were species-specific, nine occurred in both species, and one was found in hybrid populations (Fig. 1). Similarly, of the 15 mitotypes observed among *P. banksiana* populations, six were exclusive to *P. banksiana*, whereas eight and two mitotypes were shared with *P. contorta* and hybrid populations, respectively. Trees from hybrid populations carried 10 mitotypes, from which one was only found in a single hybrid population, and seven and eight were shared with *P. contorta* and *P. banksiana* populations, respectively. The highest mtDNA diversity was observed in *P. contorta* populations (*H*_T_ = 0.744; *H*_S_ = 0.484), followed by hybrid populations (*H*_T_ = 0.636; *H*_S_ = 0.390), and *P. banksiana* populations (*H*_T_ = 0.591; *H*_S_ = 0.346; Table 1). Hybrid populations (*F*_ST_ = 0.395) were also more differentiated than *P. banksiana* and *P. contorta* populations (*F*_ST_ = 0.349 and 0.282, respectively). Details about diversity parameters for each population can be found in Supplementary Table S1.

**Table 1:** Sampling description, diversity, and differentiation indices for the consolidated dataset and for each of *Pinus contorta*, *Pinus banksiana*, and the morphological hybrid group^a^

			mtDNA				cpDNA			
				
Morphological classification	Number of populations	Number of trees sampled	*nh*^b^	*H*_T_^c^	*H*_S_^d^	*F*_ST_^e^	*nh*^b^	*H*_T_^c^	*H*_S_^d^	*F*_ST_^e^
*Pinus contorta*	21	344	15	0.744	0.484	0.282	1	0	0	0
*Pinus banksiana*	23	412	14	0.591	0.346	0.349	2	0.005	0.003	0.009
Hybrid	6	169	10	0.636	0.390	0.395	2	0.468	0.166	0.907
Total	50	925	22	0.778	0.409	0.424	2	0.493	0.007	0.977

a See Supplementary Table S1 for detailed descriptive statistics per population.

b Number of mitotypes (mtDNA) and chlorotypes (cpDNA).

c Total genetic diversity.

d Within-population genetic diversity.

e Among-population differentiation.

**Figure 2:**
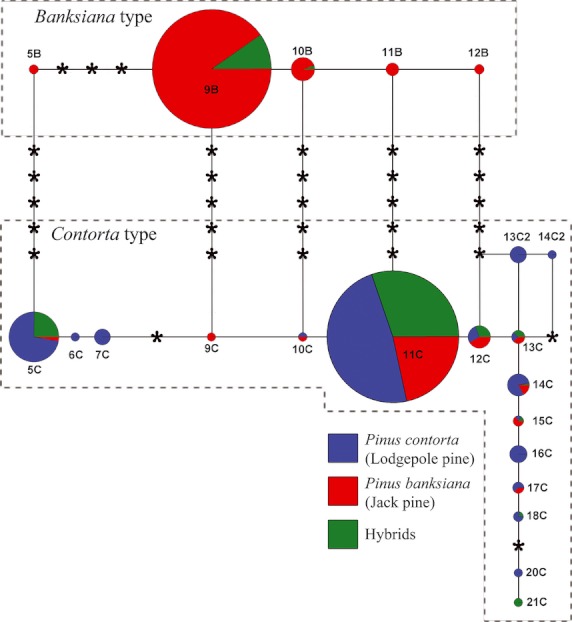
Network of the 22 mitotypes found in the *Pinus contorta–Pinus banksiana* species complex based on *nad7* intron 1 and *nad1* intron b/c polymorphisms. The size of the circle is proportional to the number of trees harboring the shown mitotype. Pie chart color corresponds to the relative frequency of tree morphological classification within each mitotype: *P. contorta*, *P. banksiana,* and hybrids. Stars are mutational steps corresponding to intermediate haplotypes not observed in the sampling. The two dashed frames separate the two mitotype groups mainly associated to each species.

The mitotype network revealed two distinct clusters that were separated by an average of six mutational steps (Fig. 2), which corresponded to the divergence observed between two *nad*1 (intron b/c) major variants and their associated low-frequency variants. The first group was predominantly associated to *P. banksiana* (*banksiana* morphological-type trees), and thus contained so-called *banskiana*-type variants (mitotype numbers followed by the letter B in Fig. 2). The second one was mostly associated to *P. contorta* (*contorta* morphological-type trees) and contained *contorta*-type variants (mitotype numbers followed by the letter C or C2 in Fig. 2). *Banksiana*-type variants were found in both *P. banksiana* and hybrid populations, whereas *contorta*-type mitotypes were found in all three population types (*P. banksiana, P. contorta,* and hybrid populations) (Fig. 2).

All four polymorphic cpDNA regions harbored one SNP each. Polymorphisms were linked, resulting in the delimitation of two distinct multi-locus chlorotypes (Table 1 and Fig. 1b). Chlorotype B was mostly associated to *P. banksiana* and almost fixed in this species (*H*_T_ = 0.005; *H*_S_ = 0.003; *f*_st_ = 0.009). Both chlorotypes (B and C) were observed in hybrid populations, whereas *P. contorta* populations were all fixed for chlorotype C (Fig. 1b). Therefore, chlorotype B was considered to be of *banksiana* type, and chlorotype C of *contorta* type. Hybrid populations presented by far the highest cpDNA diversity and population differentiation (*H*_T_ = 0.468; *H*_S_ = 0.166; *f*_st_ = 0.907).

### Geographic distribution of genetic diversity

The geographic distribution of mtDNA variation revealed the presence of mitotypes typically found in *P. contorta* populations as far east as Saskatchewan in central Canada, well within the natural range of *P. banksiana* (Fig. 1a). Globally, *P. banksiana* populations from Alberta and Saskatchewan in the near of the hybrid zone exhibited higher mtDNA diversity than those located further east. These populations from Manitoba and western Ontario were characterized by lower genetic diversity and a high abundance of mitotype 9B (Fig. 1a). Within the natural range of *P. contorta*, the highest mtDNA diversity was observed in populations from central BC and western Alberta where mitotypes 5C and 11C were the most frequent. As mentioned above, diversity at the investigated cpDNA loci was markedly split between the two species (Fig. 1b), given our specific interest to identify species-specific cytoplasmic markers to monitor interspecific gene flow. Except for one *P. banksiana* and one hybrid population from Alberta, all other stands were fixed for a single chlorotype (Fig. 1b). In *P. banksiana*, only one tree (1/412), belonging to population BANK19 from Alberta, presented the chlorotype typical of *P. contorta*.

**Figure 3:**
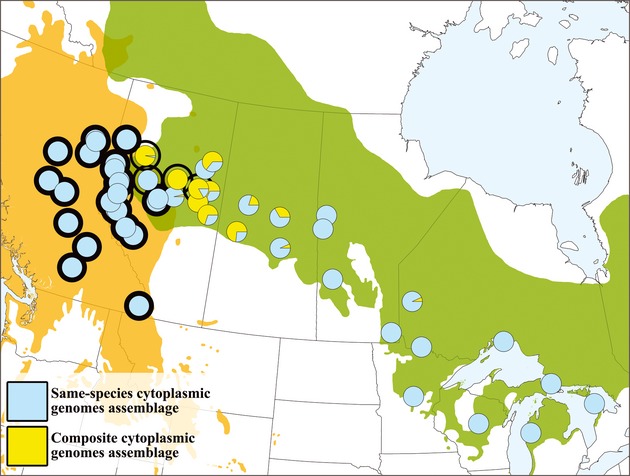
Geographic distribution of individuals presenting a same-species-specific mitotype/chlorotype assemblage (in pale blue) or a composite assemblage of mitotypes and chlorotypes from different species (in yellow). Pie charts highlighted by black circles correspond to *Pinus contorta* populations, those by white circles to hybrid populations and the other ones to *Pinus banksiana* populations. Orange background, the natural range of *P. contorta*; green background, that of *P. banksiana* (according to Little 1971).

All trees from *P. contorta* populations possessed typical *contorta* mitotype/chlorotype assemblages (Fig. 3). Trees showing composite (or hybrid) mitotype/chlorotype assemblages were found in 3 hybrid populations and 11 *P. banksiana* populations. The easternmost tree presenting such a mixed cytoplasmic genome assemblage was found in a population from Ontario where a cpDNA typical of *P. banksiana* was found in association with an mtDNA typical of *P. contorta*. Of the 106 trees carrying a mixed cytoplasmic genome assemblage, a single case of *banksiana* mitotype/*contorta* chlorotype was observed in population BANK19 from Alberta (see above), whereas all other individuals carried *contorta* mitotype/*banksiana* chlorotype combinations.

### Cluster and canonical analyses

**Figure 4:**
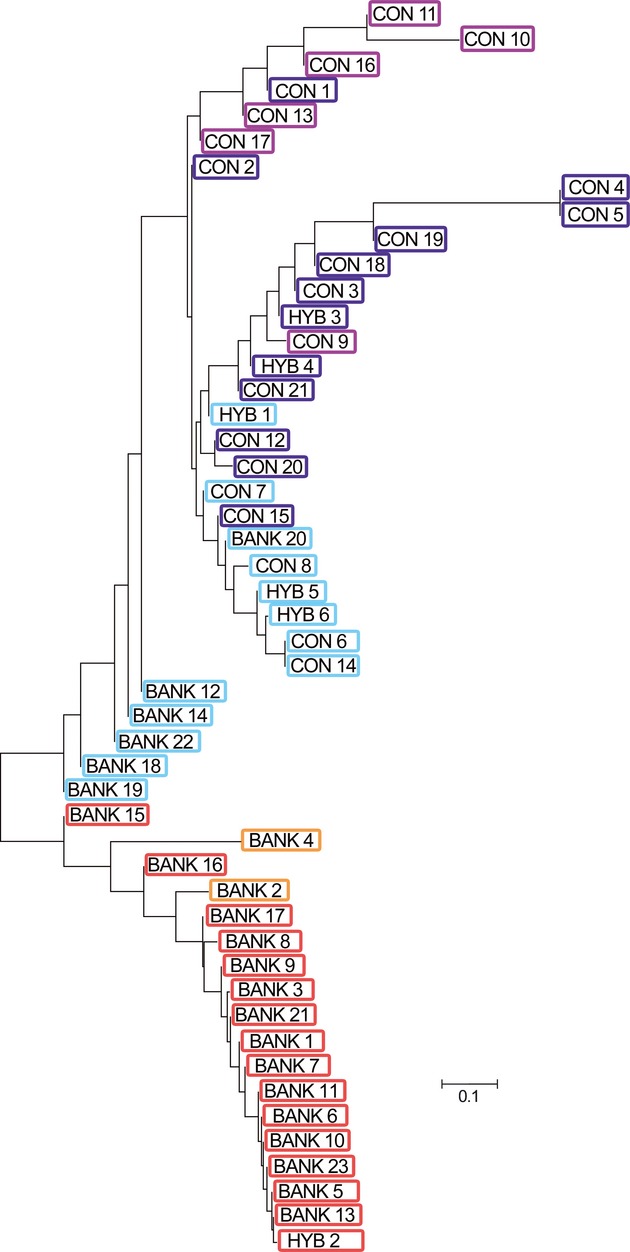
Neighbor-joining tree constructed using pairwise *F*_ST_ distances between populations based on mitotype frequencies. CON, *Pinus contorta*; BANK, *Pinus banksiana*; HYB, hybrid populations. Color frames correspond to mtDNA BAPS group membership (see Fig. 5).

The most basal branching of the NJ tree based on pairwise *F*_ST_ distances derived from mitotype frequencies separated well the two species (Fig. 4). However, five *P. banksiana* populations clustered between the two major groups. These populations were characterized by the co-occurrence of *banksiana* and *contorta* mitotypes, as previously defined from the mitotype network (Fig. 2). The *P. contorta* group was separated in two subgroups of 6 and 21 populations. These two subgroups were geographically coherent and included populations from the northwestern and south-central parts of the *P. contorta* sampling area, respectively. Five of the six hybrid populations were found in this last group. Additionally, two populations from southern BC fixed for mitotype 5C, CON4, and CON5 were found separated from the rest of the large subgroup of 21 populations.

**Figure 5:**
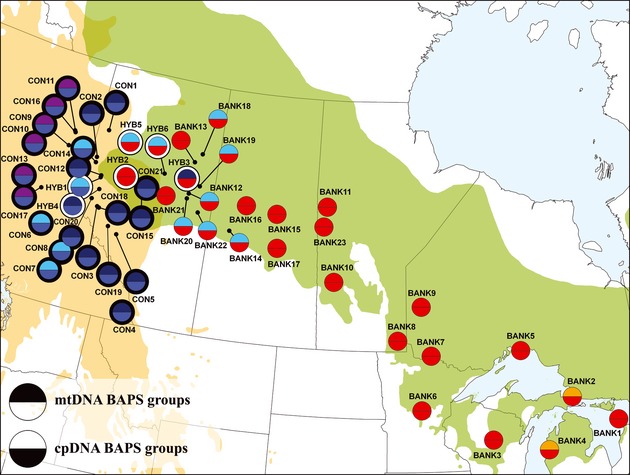
BAPS group membership of each population sampled. Pie charts highlighted by black circles correspond to *Pinus contorta* populations, those by white circles to hybrid populations, and the other ones to *Pinus banksiana* populations. Top of circles represents mtDNA BAPS groups and bottom of circles, cpDNA BAPS groups. Colors within circles correspond to BAPS group membership for each dataset. Orange background, the natural range of *P. contorta*; green background, that of *P. banksiana* (according to Little 1971).

The pattern of population grouping that resulted from the spatial Bayesian analysis (BAPS) of mitotype frequency data was very similar to the NJ dendrogram (Fig. 5). The optimal number of groups (*k*-value) was five (*f*_ct_ = 0.399). The BAPS also separated well the two species, with the exception of the “Light Blue” group (Fig. 5) that was composed by four *P. contorta*, six *P. banksiana,* and three hybrids populations located from central BC through the Saskatchewan province (*H*_S_ = 0.499, *H*_T_ = 0.579). The “Purple” group (Fig. 5) was characterized by *P. contorta* populations presenting a high diversity of mitotypes (especially 7C, 11C, 14C, and 16C; *H*_S_ = 0.685, *H*_T_ = 0.820). The “Dark Blue” group of populations (Fig. 5) contained mostly a mix of 5C and 11C mitotypes (*H*_S_ = 0.513, *H*_T_ = 0.642). Two groups of *P. banksiana* populations were also delineated: the “Orange” group (Fig. 5) was composed of populations harboring mitotype 10B (*H*_S_ = 0.360, *H*_T_ = 0.540) and the large “Red” group (Fig. 5) was characterized by populations showing a prevalence of mitotype 9B and having the lowest mtDNA diversity (*H*_S_ = 0.235, *H*_T_ = 0.247). The few incongruences noted between the Bayesian analysis and the NJ dendrogram based on *f*_st_ distances could be attributed to differences in the distribution of low-frequency mitotypes. Globally, population groupings obtained by both nonspatial and spatial analyses were coherent with the glacial lineages previously inferred for *P. banksiana* and *P. contorta* using the intron 1 region of the mtDNA gene *nad*7 (Godbout et al. 2005, 2008; see Discussion).

As expected, the BAPS analysis of cpDNA data showed a clear division between populations from the two species (Fig. 5). The two hybrid populations located in the *P. contorta* natural range were associated to the *P. contorta* group while the four remaining hybrid populations were clustered with *P. banksiana*.

The canonical redundant analysis conducted on the 14 natural populations sampled in situ in the current zone of contact between the two species identified significant associations (*P* ≤ 0.05) between cpDNA variation and the tree morphological characters defining species, as well as the ecoregions (Table 2). Such a significant relationship was not detected between cpDNA variation and ecosites. No significant relationships could be found between mtDNA variation and any of the three candidate explanatory factors.

**Table 2:** Results of Monte Carlo permutation tests for redundancy analyses (RDA) of mtDNA and cpDNA data against three explanatory factors at local sites

	mtDNA		cpDNA	
		
Explanatory factor	% constrained variance^a^	*P*-value	% constrained variance^a^	*P*-value
Species morphological classification of trees	24.5	0.156	60.4	0.010
Ecoregions	16.8	0.263	51.4	0.021
Ecosites	33.3	0.587	57.3	0.119

a Fraction of variation attributed to explanatory variables as cumulative percentages of variation explained by constrained ordination axes.

## Discussion

### Postglacial mtDNA capture by *P. banksiana*

Although the natural range of *P. contorta* does not currently extend further eastward than western Alberta, the present results show evidence of past mtDNA introgression of *P. contorta* into *P. banksiana* as far east as Saskatchewan in central Canada, which represents a distance in excess of 10 degrees of longitude or nearly 1000 km (Fig. 1). Indeed, while *P. contorta* trees carried exclusively *contorta*-associated mitotypes, almost half (44.3%) of *P. banksiana* trees from the provinces of Saskatchewan and Alberta also possessed such mtDNA background (Fig. 1). The absence of *contorta*-associated mitotypes in *P. banksiana* trees from eastern Canada (data not shown) points toward past introgression rather than ancestral polymorphism to explain the observed pattern in *P. banksiana* from Alberta and Saskatchewan. Indeed, shared ancestry would have translated into a more even geographic distribution of these mitotypes across the natural range of *P. banksiana*. This mostly unidirectional introgression pattern indicates that *P. banksiana* captured *P. contorta* mtDNA in the western part of its range. Such mtDNA capture likely occurred following the early dispersal of *P. contorta* through part of central Canada after the retreat of the continental ice sheet. The possibility that the natural range of *P. contorta* once extended further eastward than its current range was previously proposed to explain the finding of disconnected *P. contorta* stands in Alberta (Critchfield and Little 1966) and the distribution patterns of mtDNA-RFLP in Saskatchewan (Dong and Wagner 1993). Although the presence of a *contorta*-type mitotype in a population from Ontario may point to a postglacial expansion of *P. contorta* in this region, the absence of such mitotypes in Manitoba rather indicates the effect of a possible long-range dispersal of a limited number of individuals in the area (Fig. 1). The fossil record also supports this hypothesis, as *P. contorta* expanded into southern Alberta around 12 ky BP (MacDonald and Cwynar 1991), whereas the first expansion of *P. banksiana* into south Saskatchewan (∼500 km eastward) was estimated at a much later period, around 7.5 ky BP (McLeod and MacDonald 1997).

The extensive mtDNA introgression detected in the western part of the natural range of *P. banksiana* range may be explained by the demographic disequilibrium occurring when a colonizing species spreads into an area already occupied by a related species. Indeed, an early arrival of *P. contorta* before *P. banksiana* is in concordance with observations and simulations that showed a bias in the direction of introgression between two species in a zone of contact (Currat et al. 2008; Du et al. 2011). Because of the difference in species relative abundances at the time of invasion, the transfer of genes between the two species is more likely to occur from the locally abundant species (here *P. contorta*) toward the invasive one (*P. banksiana*). This trend is also most likely for the genome with least gene flow, such as for the seed-dispersed mitochondrial genome in the Pinaceae (Petit et al. 2005; Currat et al. 2008; Du et al. 2009, 2011). Assuming that *P. contorta* was well established in central Canada when *P. banksiana* first migrants reached the region, *P. banksiana* from the margin of the migration wave would have first been heavily introgressed by abundant *P. contorta*, and trees carrying this new genetic background would have then proliferated while *P. contorta* gradually declined.

The decline of *P. contorta* in western and central Canada presumably occurred between 9 and 6 ky BP during the Hypsithermal (i.e.*,* Holocene climatic optimum; Anderson et al. 1989). During this warmer period, the haploxylon/dyploxylon pollen ratio reversed in the subalpine region of southern Alberta, as a haploxylon pine (probably *Pinus flexilis*) became more abundant than a diploxylon pine (probably *P. contorta*; Anderson et al. 1989). The warmer and drier climate may have promoted the northward and eastward expansion of prairie species east of the Rocky Mountains (Williams et al. 2009). Remnants of this ecosystem can still be found in this region, as several grassland species currently present disjunct distributions (Strong and Hills 2003). Thus, *P. contorta* may have retreated to higher elevations in northern Alberta during this warmer period before it almost completely disappeared.

Putative introgression of *P. banksiana* into *P. contorta* populations has been reported using several types of characters including wood resin (Mirov 1956; Smith 1983; Pollack and Dancik 1985), cortex properties (Forrest 1980, 1981), morphological traits (Critchfield 1957; Pauly and Rudloff 1971; von Rudloff 1975; von Rudloff and Nyland 1979; Critchfield 1980; Wheeler and Guries 1987), or allozyme data (Dancik and Yeh 1983; Wheeler and Guries 1987). As these occurrences of *P. banksiana-*type characters were found to be largely scattered and randomly distributed at a large scale and low frequency among *P. contorta* populations, Critchfield (1985) proposed that the putative introgression observed in *P. contorta* populations predated the last glaciation. This interpretation is in line with our findings that *P. banksiana* did not spread west of the Rockies during the Holocene. Furthermore, although *P. banksiana* has been less studied than *P. contorta*, variable introgression has been reported among *P. banksiana* populations using nuclear markers, such as allozymes (Wheeler and Guries 1987) and random amplified polymorphic DNA (RAPD) markers (Ye et al. 2002).

### Introgression of *P. banksiana* in relation to the phylogeography of *P. contorta*

The genotyping of the highly diverse *nad7* intron 1 allowed us to determine the intraspecific lineage responsible for the early colonization of part of central Canada by *P. contorta* following the retreat of the continental ice sheet, as seen today from the introgressed mtDNA genome of jack pine populations from eastern Alberta and western Saskatchewan (“Light Blue” BAPS group, Fig 5). This group is also represented in populations of *P. contorta* located in BC, and corresponding to a glacial population that was located south of the ice sheet and west of the Rocky Mountains (Godbout et al. 2008). Indeed, based on mitotype variation, the “Light Blue” and “Dark Blue” BAPS mtDNA groups delimited in this study correspond roughly to two previously inferred *P. contorta* lineages originating from distinct glacial populations of *P. contorta* subspecies *latifolia* located south of the ice sheet on the western and eastern sides of the Rockies, respectively (Godbout et al. 2008). However, the large zone of secondary contact between these two intraspecific lineages previously observed in eastern BC and western Alberta was not clearly detected by the Bayesian analysis, possibly because the present sampling did not cover the entire range of *P. contorta* in this study or because it included populations of *P. banksiana*. Nevertheless, with the exception of two populations found at the end of two long branches in the NJ dendrogram (CON4 and CON5; Fig. 4), populations from the “Dark Blue” BAPS group were part of this large zone of secondary contact (Fig. 5). Indeed, most of these populations contained an admixture of both mitotypes (5C and 11C) typically representative of each of the two previously mentioned *P. contorta* glacial lineages. Populations from the “Purple” BAPS group of north-central BC were also part of this large zone of secondary contact, but mostly represented a mix of trees from different lineages including an additional lineage specific to *P. contorta* subspecies *contorta* typically found in western BC (Godbout et al. 2008).

According to the present results, *P. contorta* trees from the glacial lineage located east of the Rocky Mountains (Godbout et al. 2008; this study) did not participate significantly to the eastward colonization of central Canada, despite their geographic proximity to the region. Indeed, only three *P. banksiana* individuals exhibited introgression from mitotype 5C, the most typical variant of this lineage (Fig. 2). However, this lineage was presumably of smaller size than those located west of the Rockies prior to the Holocene. This hypothesis is supported by the lack of mitotype diversity observed in the two *P. contorta* populations located southwest of the zone of contact with *P. banksiana* (CON4 and CON5; Fig. 1a), confirming a previous observation based on a larger sampling of *P. contorta* in this region (Godbout et al. 2008). This hypothesis is also supported by the low *Pinus* pollen frequencies at the last glacial maximum in this region (Williams et al. 2004). Thus, at the onset of the colonization east of the Rockies, after the continental ice sheet had retreated eastward, the presumably larger size of the *P. contorta* population located west of the Rocky Mountains might have compensated for the migration delay imposed by the crossing of the mountain range when racing to colonize Alberta and Saskatchewan against the presumably smaller *P. contorta* glacial population located on the southeastern side of the range.

The eastward expansion of *P. contorta* into central Canada did not result in a decrease of mtDNA diversity, although the colonization was proposed to have occurred via a series of founding events (Cwynar and MacDonald 1987). Indeed, the introgressed *P. banksiana* trees presented seven distinct *P. contorta* mitotypes (*n* = 105; *h*_t_ = 0.569; Fig. 1). When considering the “Light Blue” BAPS group only, all mitotypes detected in *P. contorta* ssp. *latifolia* were also found in *P. banksiana* (Figs. 1 and 5). Therefore, only a few rare and infrequent mitotypes may have been lost during the species eastward migration (Fig. 2). This diversity trend suggests that colonization may have occurred through frequent long-dispersal events that would have favored a reshuffling of genes at the migration front, and thus maintained the genetic diversity (Bialozyt et al. 2006; Fayard et al. 2009).

### Asymmetry in mtDNA and cpDNA introgression: the effect of differential gene flow

The quasi-absence of cpDNA introgression between the two boreal pines contrasted with the long lasting traces of introgression observed in the mitochondrial genome (Fig. 5). This difference may be explained by the contrasted mode of dispersal of the two cytoplasmic genomes in the Pinaceae, including pines: through pollen and seeds for the paternally inherited cpDNA (Neale and Sederoff 1988) and through seeds only for the maternally inherited mtDNA (Dong and Wagner 1993). Indeed, pollen flow was shown to be theoretically several orders of magnitude larger than seed flow in pines (Ennos 1994). Several comparative studies of population differentiation between seed-only dispersed mitochondrial genomes and seed-and-pollen dispersed chloroplast, and nuclear genomes in the Pinaceae have also shown much higher population differentiation and much reduced levels of gene flow for the mitochondrial seed-dispersed genome (e.g., Burban and Petit 2003; Gamache et al. 2003; Petit et al. 2005; Gérardi et al. 2010; Godbout et al. 2010; Wei et al. 2011). Consequently, such high pollen gene flow may have rapidly resulted in the replacement of the introgressed cpDNA background by that predominantly found in the local surrounding pollen clouds. Similar asymmetric introgression of cytoplasmic genomes, though spatially more restricted, has been recently observed between coastal and interior varieties of Douglas fir (*Pseudotsuga menziesii*) in the northern part of their distributions in BC, where many trees with mixed mtDNA and cpDNA backgrounds indicated the likely capture of coastal mtDNA by an invading cpDNA lineage from the interior of the Rocky Mountains (Wei et al. 2011). A similar pattern of genome capture has also been reported between western and more eastern glacial lineages of *Picea mariana* in an area of Holocene contact in Alberta (Gérardi et al. 2010).

The canonical analysis revealed that, contrary to mtDNA, cpDNA variation within and near the zone of contact was significantly explained by the morphological classification of local trees and the ecoregion classification (Table 2), thus being well in line with species morphological differences and ecological preferences. For instance, *P. contorta* chlorotypes were predominant in the upper foothills, where *P. contorta* pollen is expected to be the most abundant, owing to the occurrence of habitats particularly favorable to this species and high local presence of this species under such conditions (Lotan and Critchfield 1990). Also, hybrid populations (except HYB3) were also fixed or nearly fixed for a single chlorotype, whether it was typical of *P. banksiana* in some populations or typical of *P. contorta* in other populations, and it matched the most abundant species in the local landscape. These various observations indicate that the differences observed among hybrid populations in their cpDNA content reflect a decisive effect of the local pollen cloud on determining the cpDNA background of trees in hybrid populations. These results are also well in line with observations on spontaneous hybridization rates between exotic and native species of poplars where the genetic origin of the local pollen cloud has been found as the most important factor to explain variation in spontaneous hybridization (Meirmans et al. 2010). These results are concordant with recent observations suggesting that genomes experiencing higher gene flow, such as the pollen and seed-dispersed chloroplast genome in the Pinaceae, are less sensitive to introgression (Currat et al. 2008; Du et al. 2009), and thus may be better suited for species delimitation (Petit and Excoffier 2009) than the seed-dispersed mitochondrial genome. In the present case, cpDNA markers were better than mtDNA markers at delimitating current species ranges and identifying the local presence of one or the other species. This trend also reflects similar conclusions at the genus level in *Picea*, another Pinaceae with different transmission and dispersal of its cytoplasmic genomes. In this genus, cpDNA and mtDNA phylogenies were not congruent and the mtDNA phylogeny appeared as a better footprint of phylogeographic history while cpDNA phylogenies would more reflect the result of repeated reticulate evolution (Bouillé et al. 2011).

Although the present set of DNA polymorphisms did not allow to separate F_1_ from advanced-generation hybrids, it is likely that most trees in hybrid populations were of the second type, because flowering times have been shown to differ between the two species, and true hybrid trees have been reported to produce a higher proportion of aborted pollen than pure *P. banksiana* or *P. contorta* trees (Critchfield 1980). A high prevalence of introgressed trees over true hybrids is frequent in tree species, as reported for the zone of contact between *Picea mariana* and *P. rubens* in eastern Canada (Perron and Bousquet 1997) and also between *Populus alba* and *P. tremula* in Europe (Lexer et al. 2005).

Hybrid populations HYB3 and BANK19 were the only ones to carry both species-specific chlorotypes together, where the typical *contorta* chlorotype was observed in 5 of 30 and 1 of 25 trees, respectively. Such a likely recent and limited incursion of *contorta* cpDNA alleles in *banksiana*-identified populations may have been caused by the prevalence of westerly winds in the region (Bartlein et al. 1998). This weak introgression trend contrasts with that observed in the same region at the intraspecific level in *Picea mariana*, where extensive capture of a central Canada mtDNA lineage by a BC cpDNA lineage was observed, a phenomenon presumably driven largely by westerly dominant winds carrying pollen over large distances (Gérardi et al. 2010). These different trends follow the expectation that scale of differences in phenology timing or variations in hybrid fitness are likely more important driving factors of gene exchange at the interspecific level than wind intensity and direction, such as between *P. contorta* and *P. banksiana*.

The present cpDNA results are to a large extent concordant with those obtained using cpDNA RFLPs (Wagner et al. 1987). However, while similarly reporting unambiguous species delimitation as in this study, Wagner et al. (1987) and Govindaraju et al. (1989) also detected new cpDNA variants in the region of contact. These new variants were proposed to be the result of either new mutations or recombination (Govindaraju et al. 1989). No such new chlorotypes were detected in the present sampling, and we did not notice recombination between the four cpDNA loci sampled, which may indicate that the new variants discovered by these authors were the result of mutation. However, evidence for recombination among cpSSR has been reported in *P. contorta* (Marshall et al. 2001) and mtDNA recombinants of recent origin have been observed in the zone of contact between *Picea mariana* and *P. rubens* (Jaramillo-Correa and Bousquet 2005).

## Conclusions

The mtDNA and cpDNA data showed marked asymmetric introgression patterns between *P. contorta* and *P. banksiana*. The noticeable presence of *P. contorta* mitotypes well within the allopatric range of *P. banksiana* suggests an ancient mtDNA capture by *P. banksiana* several thousand years ago following an early postglacial expansion of *P. contorta* in central Canada. Contrastingly, introgression at the cpDNA level was limited because of the extensive pollen gene flow from the surrounding local species over generations. Within the zone of contact between the two species where interspecific gene flow still occurs, the cpDNA background of a given population could be well predicted from the species that was locally most abundant or from the ecological characteristics of the local environment that determine the presence of either of the two species. This trend indicates that in absence of tree morphological characterization, the most dispersed cpDNA genome should be best suited to delineate boundaries between these two species, as indicated for other Pinaceae by Du et al. (2009). Some *P. banksiana* and hybrid trees from Alberta and Saskatchewan differed from allopatric *P. contorta* and *P. banksiana* populations in terms of cytoplasmic genome assemblage (Fig. 3). Except for one tree, these individuals now carry a composite organelle background where cpDNA and mtDNA were inherited from *P. banksiana* and *P. contorta*, respectively. These de novo cytoplasmic assemblages may represent a new “nested” form of genomic diversity on which natural selection may act (Gérardi et al. 2010). It indicates convincingly that even far away from glacial refugia, genetic diversity can be high and bear distinctive features not seen in ancestral lineages.

## Supporting Information

**Table S1.** Population mtDNA and cpDNA haplotype counts and diversity indices for mtDNA and cpDNA in *Pinus banksiana* and *Pinus contorta*

## References

[b1] Anderson E (1949). Introgressive hybridization.

[b2] Anderson E, Hubricht L (1938). Hybridization in *Tradescantia*. III. The evidence for introgressive hybridization. Am. J. Bot.

[b3] Anderson TW, Mathewes RW, Schweger CE, Fulton RJ (1989). Holocene climatic trends in Canada with special reference to the Hypsithermal interval. Quaternary geology of Canada and Greenland.

[b4] Anderson TM, vonHoldt BM, Candille SI, Musiani M, Greco C, Stahler DR (2009). Molecular and evolutionary history of melanism in North American gray wolves. Science.

[b5] Arnold ML (2004). Transfer and origin of adaptations through natural hybridization: were Anderson and Stebbins right?. Plant Cell.

[b6] Bartlein PJ, Anderson PM, Anderson KH, Edwards ME, Thompson RS, Webb RS (1998). Paleoclimate simulations for North America over the past 21,000 years: features of the simulated climate and comparisons with paleoenvironmental data. Quatern. Sci. Rev.

[b7] Beckingham JD, Corns IGW, Archibald JH (1996). Field guide to ecosites of west-central Alberta.

[b8] Bialozyt R, Ziegenhagen B, Petit RJ (2006). Contrasting effects of long distance seed dispersal on genetic diversity during range expansion. J. Evol. Biol.

[b9] Bouillé M, Senneville S, Bousquet J (2011). Discordant mtDNA and cpDNA phylogenies indicate geographic speciation and reticulation as driving factors for the diversification of the genus *Picea*. Tree Genet. Genomes.

[b10] Burban C, Petit RJ (2003). Phylogeography of maritime pine inferred with organelle markers having contrasted inheritance. Mol. Ecol.

[b11] Clement M, Posada D, Crandall KA (2000). TCS: a computer program to estimate gene genealogies. Mol. Ecol.

[b12] Corander J, Sirén J, Arjas E (2008). Bayesian spatial modeling of genetic population structure. Comput. Statistics.

[b13] Critchfield WB (1957). Geographic variation in Pinus contorta.

[b14] Critchfield WB (1980). Genetics of lodgepole pine.

[b15] Critchfield WB (1985). The late Quaternary history of lodgepole and jack pines. Can. J. For. Res.

[b16] Critchfield WB, Little EL (1966). Geographic distribution of the pines of the world.

[b17] Currat M, Ruedi M, Petit RJ, Excoffier L (2008). The hidden side of invasions: massive introgression by local genes. Evolution.

[b18] Cwynar LC, MacDonald GM (1987). Geographical variation of lodgepole pine in relation to population history. Am. Nat.

[b19] Dancik BP, Yeh FC (1983). Allozyme variability and evolution of lodgepole pine (*Pinus contorta* var. *latifolia*) and jack pine (*P. banksiana*) in Alberta. Can. J. Genet. Cytol.

[b20] Demesure B, Sodzi N, Petit RJ (1995). A set of universal primers for amplification of polymorphic non-coding regions of mitochondrial and chloroplast DNA in plants. Mol. Ecol.

[b21] Dong J, Wagner DB (1993). Taxonomic and population differentiation of mitochondrial diversity in *Pinus banksiana* and *Pinus contorta*. Theor. Appl. Genet.

[b22] Du FK, Petit RJ, Liu JQ (2009). More introgression with less gene flow: chloroplast vs. mitochondrial DNA in the *Picea asperata* complex in China, and comparison with other conifers. Mol. Ecol.

[b23] Du FK, Peng XL, Liu JQ, Lascoux M, Hu FS, Petit RJ (2011). Direction and extent of organelle DNA introgression between two spruce species in the Qinghai-Tibetan Plateau. New Phytol.

[b24] Ennos RA (1994). Estimating the relative rates of pollen and seed migration among plant populations. Heredity.

[b25] Excoffier L, Laval G, Schneider S (2005). Arlequin: an integrated software package for population genetics data analysis. Evol. Bioinform. Online.

[b26] Fayard J, Klein EK, Lefèvre F (2009). Long distance dispersal and the fate of a gene from the colonization front. J. Evol. Biol.

[b27] Forrest GI (1980). Geographical variation in the monoterpenes of *Pinus contorta* oleoresin. Biochem. Syst. Ecol.

[b28] Forrest GI (1981). Geographical variation in oleoresin monoterpene composition of *Pinus contorta* from natural stands and planted seed collections. Biochem. Syst. Ecol.

[b29] Gamache I, Jaramillo-Correa JP, Payette S, Bousquet J (2003). Diverging patterns of mitochondrial and nuclear DNA diversity in subarctic black spruce: imprint of a founder effect associated with postglacial colonization. Mol. Ecol.

[b30] Gérardi S, Jaramillo-Correa JP, Beaulieu J, Bousquet J (2010). From glacial refugia to modern populations: new assemblages of organelle genomes generated by differential cytoplasmic gene flow in transcontinental black spruce. Mol. Ecol.

[b31] Godbout J, Jaramillo-Correa J, Beaulieu J, Bousquet J (2005). A mitochondrial DNA minisatellite reveals the postglacial history of jack pine (*Pinus banksiana*), a broad-range North American conifer. Mol. Ecol.

[b32] Godbout J, Fazekas A, Newton CH, Yeh FC, Bousquet J (2008). Glacial vicariance in the Pacific Northwest: evidence from a lodgepole pine mitochondrial DNA minisatellite for multiple genetically distinct and widely separated refugia. Mol. Ecol.

[b33] Godbout J, Beaulieu J, Bousquet J (2010). Phylogeographic structure of jack pine (*Pinus banksiana*; Pinaceae) supports the existence of a coastal glacial refugium in northeastern North America. Am. J. Bot.

[b34] Govindaraju DR, Dancik BP, Wagner DB (1989). Novel chloroplast DNA polymorphism in a sympatric region of two pines. J. Evol. Biol.

[b35] Hewitt GM (2004). Genetic consequences of climatic oscillations in the Quaternary. Phil. Trans. R. Soc. B Biol. Sci.

[b36] Jaramillo-Correa JP, Bousquet J (2005). Mitochondrial genome recombination in the zone of contact between two hybridizing conifers. Genetics.

[b37] Jaramillo-Correa JP, Bousquet J, Beaulieu J, Isabel N, Perron M, Bouillé M (2003). Cross-species amplification of mitochondrial DNA sequence-tagged-site markers in conifers: the nature of polymorphism and variation within and among species in *Picea*. Theor. Appl. Genet.

[b38] Jaramillo-Correa JP, Beaulieu J, Khasa DP, Bousquet J (2009). Inferring the past from the present phylogeographic structure of North American forest trees: seeing the forest for the genes. Can. J. For. Res.

[b39] Jeandroz S, Bastien D, Chandelier A, Du Jardin P, Favre JM (2002). A set of primers for amplification of mitochondrial DNA in *Picea abies* and other conifer species. Mol. Ecol. Notes.

[b40] Klier K, Leoschke MJ, Wendel JF (1991). Hybridization and introgression in white and yellow ladyslipper orchids (*Cypripedium candidum* and *C. pubescens*. J. Hered.

[b41] Lexer C, Fay MF, Joseph JA, Nica M-S, Heinze B (2005). Barrier to gene flow between two ecologically divergent *Populus* species, *P. alba* (white poplar) and *P. tremula* (European aspen): the role of ecology and life history in gene introgression. Mol. Ecol.

[b42] Little EL (1971). Atlas of United States trees, volume 1, conifers and important hardwoods.

[b43] Lotan JE, Critchfield WB, Burns RM, Honkala BH (1990). *Pinus contorta* Dougl. ex. Loud. Lodgepole pine. Silvics of North America I. Conifers.

[b44] MacDonald GM, Cwynar LC (1991). Post-glacial population growth rates of *Pinus contorta* ssp. *latifolia* in western Canada. J. Ecol.

[b45] Marshall HD, Newton C, Ritland K (2001). Sequence-repeat polymorphisms exhibit the signature of recombination in lodgepole pine chloroplast DNA. Mol. Biol. Evol.

[b46] Marshall HD, Newton C, Ritland K (2002). Chloroplast phylogeography and evolution of highly polymorphic microsatellites in lodgepole pine (*Pinus contorta*. Theor. Appl. Genet.

[b47] McLeod T, MacDonald G (1997). Postglacial range expansion and population growth of *Picea mariana*
*Picea glauca* and *Pinus banksiana* in the western interior of Canada. J. Biogeogr.

[b48] Meirmans PG, Bousquet J, Isabel N (2009). A metapopulation model for the introgression from genetically modified plants into their wild relatives. Evol. Appl.

[b49] Meirmans PG, Lamothe M, Gros-Louis M-C, Khasa D, Périnet P, Bousquet J (2010). Complex patterns of hybridization between exotic and native North American poplar species. Am. J. Bot.

[b50] Mirov NT (1956). Composition of turpentine of lodgepole x jack pine hybrids. Can. J. Bot.

[b51] Moss EH (1949). Natural pine hybrids in Alberta. Can. J. Res.

[b52] Neale DB, Sederoff RR, Hanover J, Keathley DE (1988). Inheritance and evolution of conifer organelle genomes. Genetic manipulation of woody plants.

[b53] Oksanen J, Blanchet G, Kindt R, Legendre P, Minchin PR, O'Hara RB (2010). Vegan: Community ecology package. R package version 1.17-2. http://CRAN.R-project.org/package=vegan.

[b55] Pauly G, von Rudloff E (1971). Chemosystematic studies in the genus *Pinus*: the leaf oil of *Pinus contorta* var. *latifolia*. Can. J. Bot.

[b56] Perron M, Bousquet J (1997). Natural hybridization between black spruce and red spruce. Mol. Ecol.

[b57] Petit RJ, Excoffier L (2009). Gene flow and species delimitation. Trends Ecol. Evol.

[b58] Petit RJ, Duminil J, Fineshi S, Hampe A, Salvini D, Vendramin GG (2005). Comparative organization of chloroplast, mitochondrial and nuclear diversity in plant populations. Mol. Ecol.

[b59] Pollack JC, Dancik BP (1985). Monoterpene and morphological variation and hybridization of *Pinus contorta* and *P. banksiana* in Alberta. Can. J. Bot.

[b60] Rieseberg LH, Wendel JF, Harrison RG (1993). Introgression and its consequences in plants. Hybrid zones and the evolutionary process.

[b61] von Rudloff E (1975). Volatile leaf oil analysis in chemosystematic studies of North American conifers. Biochem. Syst. Ecol.

[b62] von Rudloff E, Nyland E (1979). Chemosystematic studies in the genus *Pinus* III. The leaf oil terpene composition of lodgepole *pine* from the Yukon Territory. Can. J. Bot.

[b63] Rudolph TD, Laidly PR, Burns RM, Honkala BH (1990). *Pinus banksiana* Lamb. Jack pine. Silvics of North America I. Conifers.

[b64] Rudolph TD, Yeatman CW (1982). Genetics of jack pine.

[b65] Saitou N, Nei M (1987). The neighbor-joining method: a new method for reconstructing phylogenetic trees. Mol. Biol. Evol.

[b66] Senjo M, Kimura K, Watano Y, Ueda K, Shimizu T (1999). Extensive mitochondrial introgression from *Pinus pumila* to *P. parviflora* var. *pentaphylla* (Pinaceae). J. Plant. Res.

[b67] Shafer ABA, Cullingham CI, Côté SD, Coltman DW (2010). Of glaciers and refugia: a decade of study sheds new light on the phylogeography of northwestern North America. Mol. Ecol.

[b68] Smith RH (1983). Monoterpenes of lodgepole pine xylem resin: a regional study in western United States. For. Sci.

[b69] Soltis DE, Morris AB, McLachlan JS, Manos PS, Soltis PS (2006). Comparative phylogeography of unglaciated eastern North America. Mol. Ecol.

[b70] Strong WL, Hills LV (2003). Post-Hypsithermal plant disjunctions in western Alberta, Canada. J. Biogeogr.

[b71] Taberlet P, Gielly GL, Pautou G, Bouvet J (1991). Universal primers for amplification of three non-coding regions of chloroplast DNA. Plant Mol. Biol.

[b72] Tamura K, Dudley J, Nei M, Kumar S (2007). MEGA4: Molecular evolutionary genetics analysis (MEGA) software version 4.0. Mol. Biol. Evol.

[b73] Tsumura Y, Suyama Y (1998). Differentiation of mitochondrial DNA polymophisms in populations of five Japanese *Abies* species. Evolution.

[b74] Wagner DB, Furnier GR, Saghai-Maroof MA, Williams SM, Dancik BP, Allard RW (1987). Chloroplast DNA polymorphisms in lodgepole and jack pines and their hybrids. Proc. Natl Acad. Sci. USA.

[b75] Wagner DB, Sun Z-X, Govindaraju DR, Dancik BP (1991). Spatial patterns of chloroplast DNA and cone morphology variation within populations of a *Pinus banksiana**Pinus contorta* sympatric region. Am. Nat.

[b76] Wang X-R, Tsumura Y, Yoshimaru H, Nagasaka K, Szmidt AE (1999). Phylogenetic relationships of Eurasian pines (*Pinus*, Pinaceae) based on chloroplast *rbcL*
*matK*
*rpL20-rpS18* spacer, and *trnV* intron sequences. Am. J. Bot.

[b77] Wei X-X, Beaulieu J, Khasa DP, Vargas-Hernández J, López-Upton J, Jaquish B (2011). Range-wide chloroplast and mitochondrial DNA imprints reveal multiple lineages and complex biogeographic history for Douglas-fir. Tree Genet. Genomes.

[b78] Weir BS (1996). Genetic data analysis II.

[b79] Wheeler NC, Guries RP (1987). A quantitative measure of introgression between lodgepole and jack pines. Can. J. Bot.

[b80] Whitney KD, Randell RA, Rieseberg LH (2010). Adaptive introgression of abiotic tolerance traits in the sunflower *Helianthus annuus*. New Phytol.

[b81] Williams JW, Shuman BN, Webb T, Bartlein PJ, Leduc PL (2004). Late-Quaternary vegetation dynamics in North America: scaling from taxa to biomes. Ecol. Monogr.

[b82] Williams JW, Shuman B, Bartlein PJ (2009). Rapid responses of the prairie-forest ecotone to early Holocene aridity in mid-continental North America. Global Planet. Change.

[b83] Ye TZ, Yang R-C, Yeh FC (2002). Population structure of a lodgepole pine (*Pinus contorta*) and jack pine (*P. banksiana*) complex as revealed by random amplified polymorphic DNA. Genome.

[b84] Zavarin E, Critchfield WB, Snajberk K (1969). Turpentine composition of *Pinus contorta* × *Pinus banksiana* hybrids and hybrid derivatives. Can. J. Bot.

